# Supplementary material for: A set of multi-entry identification keys to African frugivorous flies (Diptera, Tephritidae)
Source: Zookeys. 2014 Jul 24;(428):97–108. doi: 10.3897/zookeys.428.7366 (PMC4143993; doi:10.3897/zookeys.428.7366)
Supplement: Supplementary material 4 — Key to Capparimyia [file zookeys-428-097-s004.zip › SF4_ZooKeys_key to Capparimyia/lucid_player/help/help/whgdata/whlstf5.htm]

Search Words List


**<<**

**rocks**   
**root**   
**route**   
run **1** **2** **3**   
runtime **1** **2**   
  
  
s **1** **2** **3** **4** **5** **6** **7** **8** **9**   
same **1** **2** **3** **4** **5** **6** **7**   
scan **1** **2** **3**   
**scope**   
score **1** **2** **3** **4** **5**   
scored **1** **2** **3** **4** **5**   
scores **1** **2**   
scoring **1** **2**   
**screen**   
search **1** **2**   
**searches**   
**section**   
see **1** **2** **3** **4** **5** **6** **7** **8** **9**   
segments **1** **2**   
select **1** **2** **3** **4** **5**   
selected **1** **2** **3** **4** **5**   
selecting **1** **2** **3** **4**   
**selection**   
**selections**   
**sensitive**   
**separating**   
series **1** **2**   
**server**   
session **1** **2**   
**sessions**   
set **1** **2** **3** **4** **5**   
**sets**   
**setting**   
**settings**   
**shape**   
**share**   
**sheet**   
sheets **1** **2**   
short **1** **2**   
shortcut **1** **2** **3**   
shortcuts **1** **2**   
should **1** **2** **3** **4**   
show **1** **2** **3**   
shown **1** **2** **3**   
shows **1** **2**   
similar **1** **2**   
**similarly**   
**simple**   
simply **1** **2**   
since **1** **2**   
single **1** **2** **3**   
size **1** **2**   
**slec**   
**slightly**   
small **1** **2** **3**   
**smaller**   
**smallest**   
so **1** **2** **3** **4**   
solaris **1** **2** **3**   
sometimes **1** **2**   
sort **1** **2** **3** **4**   
sorted **1** **2** **3** **4**   
sorting **1** **2**   
**sorts**   
**sp1**   
**sp3**   
**sp6a**   
species **1** **2** **3** **4** **5** **6** **7**   
**specific**   
specified **1** **2** **3**   
**specify**   
specimen **1** **2** **3** **4** **5** **6** **7** **8** **9**   
**specimens**   
**splash**   
stage **1** **2**   
**standalone**   
**start**   
**started**   
**starting**   
state **1** **2** **3** **4** **5** **6** **7** **8** **9** **10** **11** **12** **13** **14**   
states **1** **2** **3** **4** **5** **6** **7** **8** **9** **10** **11** **12** **13** **14** **15** **16**   
step **1** **2** **3**   
**steps**   
**still**   
stored **1** **2**   
**storing**   
**strikingly**   
**strings**   
sub **1** **2**   
**subgroup**   
**subject**   
submenu **1** **2**   
subset **1** **2** **3**   
subsets **1** **2** **3** **4**   
**substantially**   
**successfully**   
**suggested**   
**suitable**   
sun **1** **2**   
support **1** **2**   
supports **1** **2**   
**suppose**   
**sure**   
**suse**   
**sweet**   
system **1** **2** **3** **4** **5** **6** **7**   
**systems**   
  
  
**t**   
tab **1** **2**   
tabular **1** **2**   
taxa **1** **2**   
taxonomic **1** **2**   
**technology**   
**term**   
**terminal**   
**terms**   
**tetragonal**   
text **1** **2** **3** **4**   
them **1** **2** **3** **4**   
**therefore**   
things **1** **2** **3**   
**think**   
**thorax**   
those **1** **2** **3** **4** **5** **6**   
**though**   
three **1** **2** **3**   
thumbnail **1** **2**   
thumbnails **1** **2** **3**

**>>**
